# Paranormal belief, conspiracy endorsement, and positive wellbeing: a network analysis

**DOI:** 10.3389/fpsyg.2025.1448067

**Published:** 2025-03-10

**Authors:** Neil Dagnall, Kenneth Graham Drinkwater, Andrew Denovan, Alex Escolá Gascón

**Affiliations:** ^1^School of Psychology, Manchester Metropolitan University, Manchester, United Kingdom; ^2^School of Psychology, Liverpool John Moores University, Liverpool, United Kingdom; ^3^Department of Quantitative Methods and Statistics, Comillas Pontifical University, Madrid, Spain

**Keywords:** paranormal belief, conspiracy theory endorsement, positive wellbeing, network analysis, scientifically unsubstantiated beliefs

## Abstract

Using network analysis (NA), this study examined interrelationships between advocacy of scientifically unsubstantiated beliefs (i.e., Paranormal and Conspiracy Endorsement) and positive wellbeing outcomes (i.e., Coping, Meaning in Life, Self-Esteem, and Satisfaction with Life). A total of 1,667 participants completed study measures. Analysis revealed that Paranormal Belief (PB) and Self-Esteem were central variables. Although not directly connected, common relationships existed with Search for Meaning in Life and Avoidant Coping. PB was most strongly linked (positively) with Conspiracy Endorsement, the Cognitive-Perceptual dimension of schizotypy, Search, and Avoidant Coping. Connections indicated that PB potentially mediated relationships between Schizotypy, Search, and Avoidant Coping. Self-Esteem was most strongly linked positively with the Presence of Meaning in Life, Active Coping, and Satisfaction with Life, and negatively with Avoidant Coping and Search. Network examination also revealed that Self-Esteem bridged relationships between Coping (Active and Avoidant), Meaning in Life (Search and Presence), and Satisfaction with Life. While the correlation between PB and Self-Esteem was small, the significance of these nodes suggested that their indirect interaction (through Search and Avoidant Coping) influenced factors related to positive wellbeing. This implied that the connection between PB and enhanced Self-Esteem positively impacted wellbeing. Conversely, PB associated with low Self-Esteem reflected poorer psychological health. Therefore, subsequent research should test this notion using specific belief facets.

## Introduction

Despite lacking an accepted empirical foundation and phenomena contradicting scientific principles, Paranormal Belief (PB) remains significant in contemporary Western societies (Dagnall et al., 2016; Drinkwater et al., 2021a; Drinkwater et al., 2021b). Historically, theorists have viewed supernatural beliefs as maladaptive and/or pathological (Dagnall et al., 2022a). This perspective is flawed, as it overlooks the widespread nature of PB and stems from a limited selection of outdated studies that concentrate on belief subtypes (e.g., superstition) and/or evaluate restricted populations.

At the same time, the over-generalization of negative findings has fostered the perception of believers as a uniform group despite differing levels of belief intensity and confidence that predict reduced wellbeing. This characterization is problematic because it is overly simplistic and overlooks the heterogeneous nature of PB (Dagnall et al., 2022b). Therefore, the degree to which PB is (mal) adaptive varies as a function of scores on affiliated factors. This is particularly true regarding the co-occurrence of cognitive-perceptual constructs such as schizotypy (Denovan et al., 2018), manic-depressive experiences (Drinkwater et al., 2024), and transliminality (Dagnall et al., 2022a; Dagnall et al., 2022c).

From this perspective, the interaction between PB and cognitive-perceptual factors is crucial since it influences information processing. PB arises from a limited appreciation of scientific wisdom and flawed reasoning (Lawrence and Peters, 2004; Dagnall et al., 2007). Specifically, a preference for subjective (vs. objective) evidence (Williams et al., 2022). In light of this, some theorists consider PB to be a form of sub-clinical psychosis (e.g., Unterrassner et al., 2017), with beliefs acting as everyday delusions (Irwin et al., 2012a; Irwin et al., 2012b). This perspective aligns with the observation that core features of PB resemble attenuated psychotic symptoms (i.e., ideas of reference, odd beliefs, magical thinking, and unusual perceptual experiences).

Recent research supports the notion that the interaction between PB and cognitive-perceptual personality factors predicts wellbeing rather than credence itself. One widely studied factor is schizotypy. Theorists working with general populations view schizotypy as a multidimensional personality construct that encompasses normal variation in a range of cognitive-perceptual, interpersonal, and disorganized traits associated with susceptibility to psychosis (Claridge, 1997). This definition recognizes that schizotypy relates to both adaptive and maladaptive psychological functioning. For example, Dagnall et al. (2024) discovered that interactions between levels of PB and schizotypy were linked to differences in presence and search for meaning in life. At the variable level, PB showed a positive correlation with both presence and search, while schizotypy was positively correlated with search but negatively correlated with presence.

Meaning in life refers to the presence of purpose and the search for significance in existence (Steger, 2009). Prior research has robustly linked the balance between presence and search to reduced suffering, improved relationships, and greater life satisfaction (Steger, 2017). Thus, a realized search facilitates presence (Newman et al., 2018), while a frustrated search indicates an absence of purpose (Russo-Netzer and Icekson, 2023). This interplay is significant because presence and search are orthogonal and predict health outcomes differently. Presence aligns with positive effects (e.g., flourishing, optimal functioning, and mental health, Seligman, 2011), whereas search is associated with negative factors (e.g., stress and emotional discomfort) (Newman et al., 2022). Additionally, recent studies have shown that PB, in the absence of cognitive-perceptual factors linked to psychopathology (e.g., transliminality, hypersensitivity to psychological material; Dagnall et al., 2022a), is not related to negative wellbeing outcomes (e.g., stress and somatic complaints) (Drinkwater et al., 2024).

Another factor that has clouded the relationship between PB and wellbeing is the tendency of researchers to categorize supernatural belief alongside the endorsement of conspiracy theories (CT). This pairing arises from the observation that PB and CT positively correlate and share similar affiliations with various other variables. For instance, the need for cognitive closure (i.e., the desire for certainty and dislike of ambiguity, Kruglanski and Webster, 1996; Imhoff and Bruder, 2014), distrust of authority and institutions (Bruder et al., 2013; Leman and Cinnirella, 2007), feelings of powerlessness or social alienation (Goertzel, 1994; Imhoff and Bruder, 2014), and high openness to experience (Bruder et al., 2013; Imhoff and Bruder, 2014).

Additionally, PB and CT share common characteristics. Specifically, both constructs are epistemically unwarranted beliefs (i.e., they exceed the totality of evidence/knowledge) (Lobato et al., 2014), are associated with magical thinking (Oliver and Wood, 2018), and reflect ontological confusion (Rizeq et al., 2021). They arise from mentalizing biases that lead to the inappropriate classification of distinctive properties of superordinate categories (i.e., mental and physical, animate and inanimate, and living and lifeless) (Dyrendal et al., 2021; Lindeman et al., 2015).

Focusing on the commonalities between PB and CT has led to the oversight of conceptual differences that affect the formation of relationships around wellbeing. In this context, it is important to note that, although moderately to highly correlated, PB and CT share only 9–25% of variance (Drinkwater et al., 2012; Dagnall et al., 2017). Furthermore, when positioned within the taxonomy of rational thinking problems developed by Stanovich et al. (2008), PB and CT reflect distinct failures in rational thought. Specifically, PB corresponds with mindware gaps (i.e., flawed understanding of scientific knowledge) and CT with contaminated mindware (i.e., problematic data that hinders evaluation, encourages egocentric thought, and fosters maladaptive conditioned beliefs) (Bensley et al., 2020).

Consistent with this divergence, PB and CT relate differently to social worldviews (i.e., life representations). PB is positively associated with the belief in a just world (i.e., the notion that people get what they deserve) and negatively linked to a competitive worldview (i.e., the perception of others as cut-throat rather than cooperative) (Grigoryev and Gallyamova, 2023). In contrast, CT is positively related to a dangerous worldview (i.e., an inclination to sense risk and threat), a competitive worldview, and a zero-sum game belief (i.e., social/relationship cynicism). The literature reports additional differences, such as PB (vs. CT) believers demonstrating greater environmental control (Van Prooijen and Acker, 2015).

### The present study

Noting the lack of studies examining the wellbeing-related benefits of PB, this paper investigates the relationships between supernatural beliefs and factors related to positive wellbeing (i.e., coping, meaning in life, self-esteem, and life satisfaction). In line with prior research (see Betsch et al., 2021; Parra and Corbetta, 2014), the researchers expected that PB would have a positive association with health-promoting outcomes. For instance, Kennedy and Kanthamani (1995) found that increased PB strengthened connections to others and raised happiness, confidence, optimism, and meaning. Furthermore, Irwin (2009) suggested that PB enhanced life satisfaction by mediating happiness orientations.

To examine whether PB and CT differ in adaptivity (i.e., their associations with factors linked to positive wellbeing), the researchers included a measure of conspiratorial ideation. While PB has been associated with positive effects, prior research connects CT to negative outcomes such as anxiety, fear, and distrust, which can undermine wellbeing (Van Prooijen and Douglas, 2017). Thus, while PB reflects affirming ideations, including hope and a sense of universal meaning or connectedness, CT represents negative, antagonistic cognitions that challenge social conventions and institutions (Imhoff and Bruder, 2014). Concomitant with the opposing nature of these constructs, the authors expected that PB (vs. CT) would be more strongly related to positive wellbeing outcomes. Despite being highly correlated, they noted that PB, CT, and schizotypy differ in their relationships with established wellbeing outcomes such as life satisfaction and Self-Esteem (see Dagnall et al., 2024). Acknowledging this and the potential mediating effect of schizotypy on scientifically unverified beliefs, the researchers included schizotypy in the current study.

The researchers used network analysis (NA) to explore the relational properties among scientifically unverified beliefs (PB and endorsement of CT), schizotypy, and factors associated with positive wellbeing. The authors chose NA because the technique isolates interacting and/or mutually reinforcing variables. Concurrently, NA, through the simultaneous assessment of relationship patterns, specifies the influence of multiple constructs within a proposed, hypothesized system (Levinson et al., 2017). Thus, the use of NA in the present study aided in identifying complex, conceptually significant relationships. This was beneficial since the investigation was exploratory. Furthermore, considering variable interconnections, often obscured by traditional variable-centered approaches that focus on unidirectional or linear relationships, expanded previous scholarly studies.

Another reason for using NA was that the technique allowed the researchers to account for the conceptual overlap between PB, CT, and schizotypy. This aspect was significant because, although these constructs are distinct, they function interdependently. Therefore, changes in one variable tend to affect the others. NA illustrated reciprocal relationships by representing variables as nodes, associative strengths as links, and edges as pairwise conditional associations between factors (Borsboom et al., 2021).

Simultaneously, NA identifies constructs that significantly influence the proposed network. For example, NA can reveal whether schizotypy serves as a central node linking PB and CT while mediating relationships with positive wellbeing. Furthermore, the application of NA facilitated the integration of positive wellbeing factors. Specifically, it enabled a simultaneous examination of how wellbeing interacts with potentially less adaptive constructs (i.e., CT and schizotypy). Notably, NA identifies positive wellbeing variables in the network that protect individuals from maladaptive tendencies by moderating their effects or redirecting cognitive and emotional resources toward constructive outcomes. These insights are invaluable because they can guide the design of targeted interventions to promote psychological health (Valente, 2012; Heshmati et al., 2020).

Another reason for using NA was the technique’s capability to evaluate the strength and directionality of relationships and identify clusters of closely related variables. Such features are vital when examining dynamic and context-dependent phenomena, such as belief systems and psychological health variables.

In summary, this paper used NA because the technique provided a nuanced, multidimensional framework for investigating the complex interplay among scientifically unverified beliefs (PB and CT), schizotypy, and positive wellbeing. Specifically, the adoption of NA allowed the researchers to holistically examine and identify patterns and mechanisms that would not have surfaced through traditional variable-centered approaches (Heshmati et al., 2020). Indeed, the advantages of NA explain why scholars in psychopathology and psychology have adapted the technique from computer science. In the health domain, symptoms represent key elements of the conditional system, and the network serves as a means for simultaneously clarifying varying effects (i.e., type: positive and negative; connectedness: distal and proximal; direction: unidimensional and reciprocal). Relationship strengths also indicate the level of psychological adjustment (i.e., the condition’s nature, expression, and course) (Levinson et al., 2017; Jones et al., 2018).

## Materials and methods

### Participants

The sample included 1,667 participants (Mage = 47.79, SD = 12.46, range 18–79); 871 males (Mage = 49.01, SD = 11.81, range 18–79), 783 women (Mage = 46.53, SD = 12.98, range 18–73), two trans men (Mage = 32.0, SD = 9.89, range 25–39), three trans women (Mage = 56.66, SD = 7.50, range 49–64), seven non-binary individuals (Mage = 37.42, SD = 15.60, range 18–59), and one participant who preferred not to disclose their gender (Age = 59.00). Regarding educational qualifications, the sample included 330 participants with pre-degree education, 480 with undergraduate degrees, 317 with postgraduate degrees, 350 with vocational qualifications, and 190 with professional qualifications.

The researchers were recruited through Bilendi, a participant panel provider known for delivering high-quality data (Fladerer and Braun, 2020). This approach allowed the researchers to access a broad age range, maintain gender balance, and generate responses comparable to those obtained through traditional methods (Kees et al., 2017). The online survey platform Qualtrics hosted the survey, which participants accessed via a web link. Bilendi distributed the link to panel members who had agreed to participate in the surveys. The researchers requested a United Kingdom-based, gender-balanced sample and specified that participants must be at least 18 years old.

### Measures

The study utilized established self-report measures, which required participants to respond to statements by completing corresponding response scales.

### Belief

#### Revised paranormal belief scale (RPBS)

The RPBS (Tobacyk, 2004) consists of 26 items that sample seven primary dimensions of supernatural belief (i.e., traditional religious beliefs, psi, witchcraft, spiritualism, superstition, extraordinary life forms, and precognition) (Dagnall et al., 2010; Drinkwater et al., 2017). Each item is presented as a statement (e.g., ‘I believe in God’), and participants indicate their level of agreement on a 7-point Likert scale, from 1 = Strongly Disagree to 7 = Strongly Agree. Before statistical analysis, the researchers, in line with Irwin (2009), converted scores from 0 to 6. Thus, total scores ranged from 0 to 156, with higher scores reflecting a greater belief in the paranormal. In this study, omega reliability was good, *ω* = 0.96.

#### Generic conspiracist beliefs scale short (GCB-5)

The GCB-5 (Kay and Slovic, 2023) is a shortened version of the 15-item Generic Conspiracist Beliefs Scale (GCBS, Brotherton et al., 2013). It includes the highest loading items from each of the five GCBS dimensions: government malfeasance, extraterrestrial cover-up, malevolent global conspiracy, personal wellbeing, and information control. Researchers created this condensed instrument for use in extensive test batteries. Participants indicate their level of agreement on a 5-point Likert-type scale (1 = not true to 5 = definitely true). Total scores ranged from 5 to 25, with higher scores reflecting agreement with generic conspiracist beliefs. The current study demonstrated good internal reliability, *ω* = 0.87.

### Schizotypy

#### Schizotypal personality questionnaire-brief (SPQ-B)

The SPQ-B (Raine and Benishay, 1995) is a 22-item tool designed to assess the presence of schizotypal features and personality disorders in general populations. Participants answer items (e.g., ‘Some people think that I am a very bizarre person’) using a binary scale (0 = No and 1 = Yes). The SPQ-B includes three dimensions: Cognitive-Perceptual Deficits (eight items), Interpersonal Deficits (eight items), and Disorganization (five items).

The Cognitive-Perceptual subscale evaluates the presence of characteristics similar to the positive symptoms of schizophrenia (i.e., unusual perceptual experiences, odd beliefs, magical thinking, ideas of reference, and paranoid ideation). The Interpersonal subscale broadly aligns with negative indicators of schizophrenia (i.e., constricted affect, social anxiety, paranoia, and lack of close friends). The Disorganized subscale corresponds to the thought disorder and bizarre behavior dimensions of schizophrenia (i.e., odd speech and unusual behavior), as noted by Arndt et al. (1991). In addition to subscale scores, the SPQ-B generates total scores that range from 0 to 22, with higher scores indicating greater levels of schizotypy. The subscales in this study demonstrated satisfactory reliability: Cognitive-Perceptual *ω* = 0.78, Disorganized *ω* = 0.78, Interpersonal *ω* = 0.82.

### Factors allied to positive wellbeing

#### Brief-COPE

The Brief-COPE (Carver, 1997) assesses both effective and ineffective coping responses to stressors. In this context, coping refers to strategies aimed at alleviating distress caused by negative life experiences. In the present study, coping was categorized into active (12 items, e.g., ‘I’ve been taking action to try to make the situation better’) and avoidant (12 items, e.g., ‘I’ve been refusing to believe that it has happened’) approaches (Eisenberg et al., 2012). Participants provided their responses using a 4-point Likert-type scale ranging from 1 (‘I usually do not do this’) to 4 (‘I usually do this a lot’). Both subscales yielded scores from 12 to 48, with higher scores indicating a greater reliance on active and Avoidant Coping. The present study demonstrated good reliability, with Active Coping *ω* = 0.92 and Avoidant Coping *ω* = 0.90.

#### Meaning in Life Questionnaire (MLQ)

The MLQ (Steger et al., 2006) is a 10-item tool that assesses both the presence of meaning (5 items, ‘I have a good sense of what makes my life meaningful’) and the search for meaning (5 items, ‘I am searching for meaning in my life.’). “Presence” refers to the extent to which participants feel their lives have a purpose, while “search” indicates how actively participants seek to find or enhance this sense of purpose. Participants respond using a 7-point Likert-type scale, ranging from 1 (‘absolutely untrue’) to 7 (‘absolutely true’). Total scores for both subscales ranged from 5 to 35, with higher scores indicating greater presence and search. Both subscales demonstrated strong internal consistency, with Presence *ω* = 0.91 and Search *ω* = 0.93.

#### Rosenberg Self-Esteem Scale (RES)

The RES (Rosenberg, 1965) is a 10-item tool designed to assess global Self-Esteem, which reflects feelings of self-worth and self-acceptance. Participants respond to each statement (e.g., ‘I feel that I have a number of good qualities’) using a 4-point Likert scale, where 1 means ‘Strongly Disagree,’ and 4 means ‘Strongly Agree.’ Total scores ranged from 10 to 40, with higher scores indicating greater levels of Self-Esteem. The RES demonstrated good reliability in the current study, with an *ω* of 0.91.

#### Satisfaction with Life Scale (SWLS)

The SWLS (Diener et al., 1985) is a 5-item instrument that measures the cognitive component of subjective wellbeing. It affords an integrated assessment of life satisfaction (e.g., ‘In most ways my life is close to my ideal’). Participants record responses on a 7-point Likert-type scale, ranging from 1 (‘Strongly Disagree’) to 7 (‘Strongly Agree’). Scores range from 5 to 35, with higher values indicating greater life satisfaction. This study had good reliability, *ω* = 0.94.

The scales used in the present study possessed attested psychometric properties (i.e., validity and reliability): RPBS (Drinkwater et al., 2017; Tobacyk, 2004), GCB-5 (Dagnall et al., 2023; Kay and Slovic, 2023), SPQ-B (Raine and Benishay, 1995), Brief-COPE (Carver, 1997), MLQ (Steger et al., 2006), RES (Rosenberg, 1965), and SWLS (Diener et al., 1985).

### Procedure

Upon receiving the weblink, participants accessed the information sheet detailing the study. Those wishing to participate then proceeded to the online survey. The initial section included a brief demographic survey requesting age, preferred gender, and occupation. Participants then moved on to the measures. The order of items was rotated among participants to reduce potential order and carryover effects. Instructions directed participants to navigate the sections independently and read items thoroughly. The researchers utilized counters to minimize common method variance. This was necessary because the investigators gathered data at a single point in time (i.e., cross-sectional). To distinguish constructs, instructions in each section emphasized the uniqueness of each scale (Spector, 2019). This created psychological distance between measures and encouraged participants to engage in thoughtful reflection. Furthermore, to lower the chances of social desirability and evaluation apprehension, the guidelines clarified that there were no right or wrong answers and that responses should reflect personal preferences (Krishnaveni and Deepa, 2013). At the end of the survey, participants received a debrief about the study.

### Ethics statement

The Health and Education Research Ethics and Governance Committee at Manchester Metropolitan University granted ethical approval (Project ID, 47784).

### Data analysis plan

After data screening, evaluation of descriptive statistics, and consideration of Pearson correlations, the researchers estimated a network (using JASP). This network included PB, Conspiracy Endorsement (CT), Schizotypy, Meaning in Life, Coping, Self-Esteem, and Satisfaction with Life. The analysis utilized the graphical least absolute shrinkage and selection operator based on the Extended Bayesian Information Criterion (i.e., the EBICglasso; Chen and Chen, 2008; Friedman et al., 2008). A tuning parameter of 0.5 was applied to produce a parsimonious network.

Betweenness, closeness, and strength represent variable centrality. Betweenness indicates the significance of bridging and connecting (i.e., how many nodes lie on paths between other nodes). Closeness reflects the distance between nodes, where shorter distances indicate greater influence. Strength pertains to the total of paths leading to and from a node (Bryant et al., 2017). To assess network stability, the analysis utilized a bootstrap 95% confidence interval (CI), which was calculated for network edges based on 1,000 resamples (Epskamp et al., 2018).

## Results

### Descriptive statistics and correlations

Data screening (see Appendix S1) indicated satisfactory skewness and kurtosis (i.e., within −2 and + 2 standard deviations) (Hair et al., 2022). The presence of multiple correlations necessitated controlling for the familywise error rate. This required a sequential approach to interpreting results, where the ranking of *p*-values was conducted alongside critical *p*-values (Benjamini and Hochberg, 1995). Comparisons used the 0.05 significance threshold.

Using the Benjamini and Hochberg (1995) procedure, PB and CT correlated positively with Schizotypy (Cognitive-Perceptual, Interpersonal, and Disorganized), Coping (more strongly with Avoidant than Active), and negatively with Self-Esteem. Regarding Meaning in Life, PB showed a positive correlation with both Presence and Search, while CT was only positively related to Search (Table 1). For coping, Active was positively associated with Self-Esteem and Life Satisfaction, whereas Avoidant Coping had a negative association. The Bayesian correlation test confirmed the strength of most associations, with log-transformed Bayes Factors (log (BF_10_)) exceeding 3.

**Table 1:** Correlations among study variables.

Variable	1	2	3	4	5	6	7	8	9	10	11
1. Paranormal Belief											
log(BF_10_)											
2. Conspiracy Endorsement	0.60**										
log(BF_10_)	366.68										
3. Cognitive-Perceptual	0.56**	0.48**									
log(BF_10_)	307.85	211.92									
4. Interpersonal	0.16**	0.18**	0.47**								
log(BF_10_)	18.84	23.06	199.08								
5. Disorganized	0.25**	0.25**	0.58**	0.60**							
log(BF_10_)	49.51	49.94	332.19	369.38							
6. Meaning in Life Presence	0.10**	0.01	−0.03	−0.30**	−0.20**						
log(BF_10_)	5.42	−3.47	−2.81	72.59	28.74						
7. Meaning in Life Search	0.42**	0.34**	0.38**	0.21**	0.25**	−0.04					
log(BF_10_)	160.35	97.31	127.46	33.01	50.25	−2.47					
8. Active Coping	0.09**	0.06*	0.14**	−0.05*	−0.01	0.39**	0.17**				
log(BF_10_)	3.94	−0.39	11.91	−1.21	−3.46	131.64	20.09				
9. Avoidant Coping	0.40**	0.32**	0.40**	0.26**	0.37**	−0.11**	0.40**	0.10*			
log(BF_10_)	144.90	83.24	144.66	52.66	117.78	5.99	141.95	0.63			
10. Self-Esteem	−0.16**	−0.13**	−0.25**	−0.42**	−0.37**	0.61**	−0.28**	0.32**	−0.40**		
log(BF_10_)	17.09	11.43	50.29	155.65	120.70	385.32	61.86	87.83	137.25		
11. Satisfaction with Life	0.01	−0.05*	−0.14**	−0.30**	−0.17**	0.61**	−0.16**	0.23**	−0.09**	0.55**	
log(BF_10_)	−3.48	−1.13	13.62	76.94	21.85	380.94	18.90	43.39	2.64	297.78	

*p < 0.05; **p < 0.001 (also less than the Benjamini and Hochberg adjusted critical *p*- values).

### Network analysis

**Figure 1:**
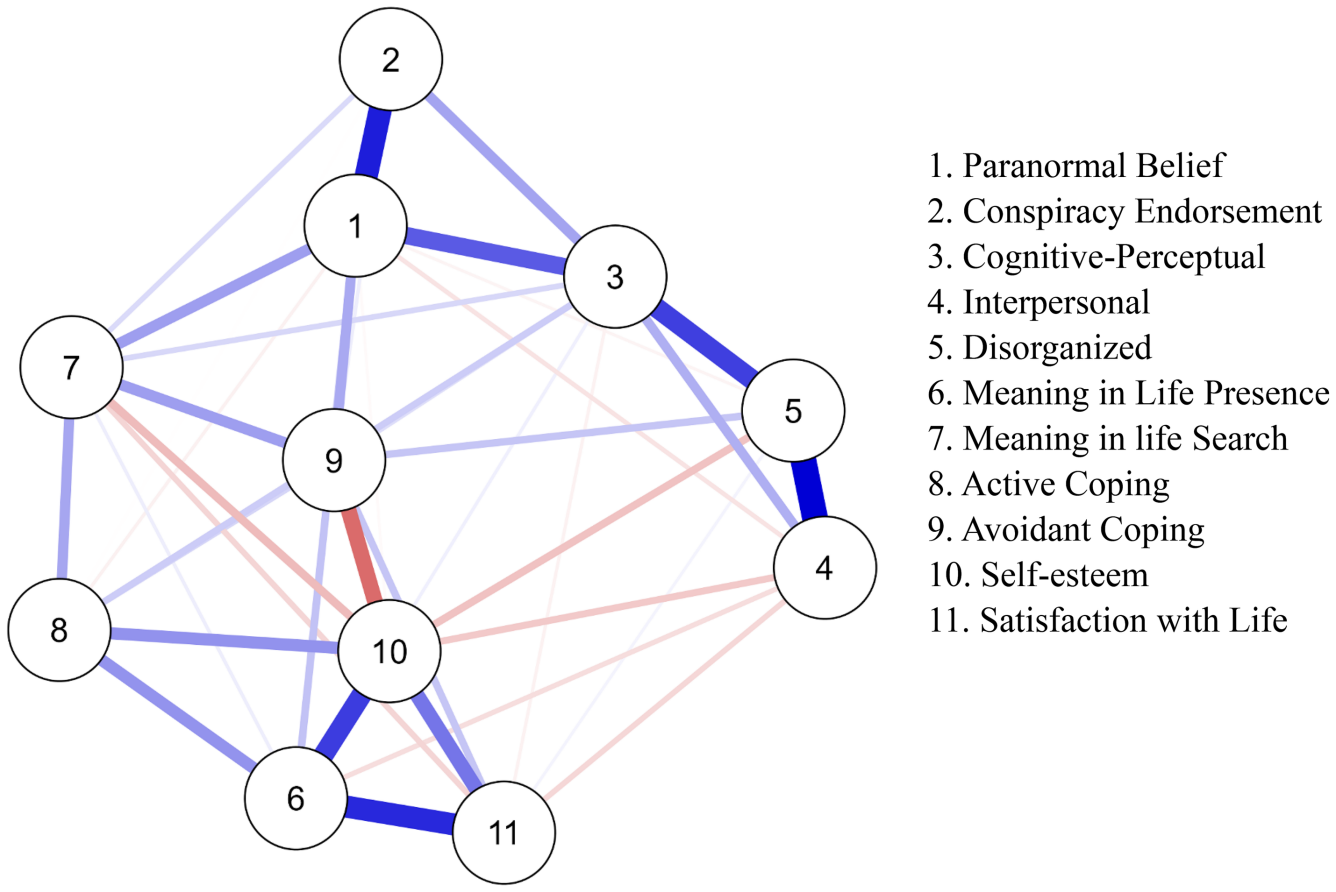
Network analysis of paranormal beliefs, CT, schizotypy, meaning in life, coping, and wellbeing (EBICglasso model). Blue lines indicate positive associations, while red lines indicate negative associations. Thicker lines represent stronger associations.

Figure 1 shows the EBICglasso network, which includes 11 nodes and 40 non-zero edges. Blue and red edges indicate positive and negative relationships, while thicker edges signify stronger links. The network displayed interconnectivity, with strong edges present between PB, CT, and Schizotypy (especially for the Cognitive-Perceptual dimension). PB also connected with to Search and Avoidant Coping, with the latter negatively correlating with Self-Esteem. Additionally, strong edges existed between Active Coping, Presence, Self-Esteem, and Satisfaction with Life.

The weights matrix (Appendix S2) confirmed these outcomes, showing a strong link between PB and CT. A positive relationship existed between these constructs and the Cognitive-Perceptual subscale. A positive link emerged between PB, Search, and Avoidant Coping; however, this connection was weaker for CT. Active Coping linked positively with Presence, Search, and Self-Esteem. Avoidant Coping linked positively with Search and Presence but negatively with Self-Esteem. Search showed negative relationships with Self-Esteem and life satisfaction, while Presence exhibited a positive relationship. For edge weight accuracy (calculated using bootstrap 95% non-parametric CIs), refer to Appendix S3. As indicated by narrow CIs and the majority not crossing zero, network edges were reliable.

**Figure 2:**
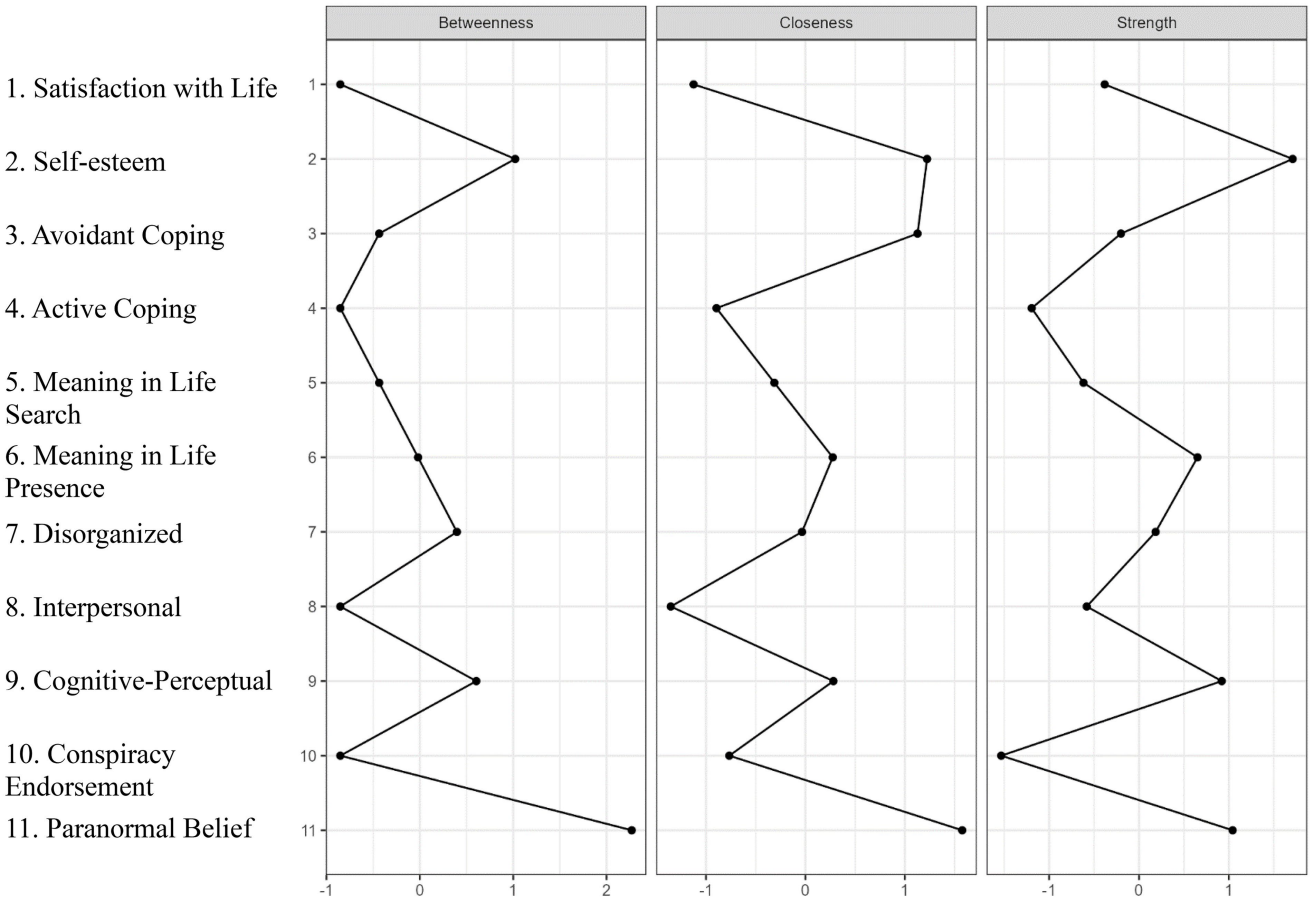
Centrality plots depicting betweenness, closeness, and strength for each node (variable). The values are standardized z-scores that indicate the relative importance of nodes in the networks.

Table 2 presents standardized estimates of betweenness, closeness, and strength centrality indices. To aid in interpretation, Figure 2 illustrates the centrality plots. The nodes with the highest strength centrality were Self-Esteem, Presence, Cognitive-Perceptual, and PB. The nodes with the lowest strength centrality were Active Coping and CT. PB and Self-Esteem exhibited the greatest betweenness centrality, while Satisfaction with Life, Active Coping, Interpersonal, and CT had the lowest. Self-Esteem, Avoidant Coping, and PB achieved the highest closeness centrality.

**Table 2:** Centrality measures.

Variable	Betweenness	Closeness	Strength
Paranormal belief	2.27	1.58	1.04
Conspiracy endorsement	−0.85	−0.77	−1.53
Cognitive-perceptual	0.61	0.28	0.92
Interpersonal	−0.85	−1.35	−0.58
Disorganized	0.40	−0.03	0.19
Meaning in life presence	−0.02	0.28	0.65
Meaning in life search	−0.44	−0.31	−0.62
Active coping	−0.85	−0.89	−1.19
Avoidant coping	−0.44	1.13	−0.20
Self-esteem	1.02	1.22	1.71
Satisfaction with life	−0.85	−1.13	−0.38

Based on the associations and centrality of PB and Self-Esteem, these constructs meaningfully bridged/connected other constructs within the network. Specifically, PB linked CT, Cognitive-Perceptual, Search, and Avoidant Coping. Self-esteem, in turn, linked Avoidant Coping, Active Coping, Search, Presence, and Satisfaction With Life (Figure 1).

However, the RPBS distinguishes between classical PBs and religious PBs (Williams and Roberts, 2016). Consequently, a strict focus on the RPBS at a composite level may have introduced error. Therefore, a second analysis focused on TRBs instead of the full RPBS to determine the accuracy of the initial conclusions. This served as a robustness test of the network and the position/role of PB. The results aligned with those of the full RPBS, showing a positive link with CT, Cognitive-Perceptual, Search, and Avoidant Coping (see Appendix S4). Additionally, TRB was central to these variables. The relationships with variables such as CT and Cognitive-Perceptual were weaker, possibly due to the fewer perceptual-focused items.

## Discussion

In the network, the central variables were PB and Self-Esteem. Although not directly linked, there were common relationships between Meaning in Life (Search) and Avoidant Coping. PB most strongly linked (positively) with CT, Cognitive-Perceptual, Search, and Avoidant Coping. The pattern of connections indicated that PB bridged (and potentially mediated) relationships between Schizotypy, Search, and Avoidant Coping. Self-Esteem most strongly linked (positively) with Presence, Active Coping, and Satisfaction with Life, and negatively with Avoidant Coping and Search. The network indicated that Self-Esteem bridged relationships between Coping, Meaning in Life, and Satisfaction with Life.

While the correlation between PB and Self-Esteem was small (Gignac and Szodorai, 2016), the importance of these nodes suggested that their indirect interaction (via Search, and Avoidant Coping) influenced factors allied to positive wellbeing. This idea aligns with Maraldi (2014), who found that low Self-Esteem in childhood was associated with increased passivity and avoidance of personal responsibility. Also, that these factors were concomitant with perceived lack of personal control and increased reliance on external agencies, such as paranormal forces and/or agents. In the context of this study, this suggests that PB linked to enhanced Self-Esteem is affiliated with positive wellbeing, whereas PB associated with low Self-Esteem is related to reduced or poorer psychological health.

This assertion aligns with subsequent investigations that produced conflicting outcomes (Pérez Navarro and Martínez Guerra, 2020). While some studies indicate an inverse relationship between PB and Self-Esteem, others report a positive correlation (Fitzpatrick and Shook, 1994). The former suggests that supernatural belief empowers individuals with low Self-Esteem, while the latter implies that PB reflects a grandiose sense of personal importance and uniqueness (Pérez Navarro and Martínez Guerra, 2020). Given the inconsistent nature of prior research and the exploratory focus of this paper, future studies should further evaluate how the interrelationship between PB and Self-Esteem impacts wellbeing. This is particularly significant since, in the present study, PB and Self-Esteem are connected differently to Schizotypy, Meaning in Life, Coping, and Life Satisfaction. Specifically, Self-Esteem linked directly to Active Coping (positive), Meaning in Life (presence, positive; search, negative), and Avoidant Coping (negative). Satisfaction with Life linked directly with Presence (positive) and Search (negative).

Regarding CT, this strongly connected with PB (the bridging node) and directly linked to Cognitive-Perceptual and Search. Similarly, Search linked with Avoidant Coping, although weaker with Cognitive-Perceptual. Examining the correlations for PB and CT, both constructs displayed similar relationships with Schizotypy, Search, Active Coping, Avoidant Coping, and Self-Esteem. The analysis indicated differences on Presence (PB showed a small positive association while CT showed no relationship) and Satisfaction with Life (PB showed no relationship while CT exhibited a small negative association).

Differences on Meaning in Life are conceptually significant because research has shown that these two dimensions relate to different health outcomes. Presence is connected to positive factors such as life satisfaction and is inversely linked to negative aspects (e.g., depression) (Steger et al., 2006). Search is associated with decreased wellbeing and negative emotions (e.g., sadness and rumination) (Dakin et al., 2021). In the case of PB, Presence mitigates the negative outcomes linked to Search (Newman et al., 2018). The lack of an association between CT and Presence suggests that this does not apply to belief in conspiracies. This possibly explains why, despite the relationship being weak, CT was negatively associated with Satisfaction with Life.

## Limitations

Since the inclusion of other psychological constructs may influence the system, readers should limit conclusions to the variables considered within the proposed network. Therefore, future investigations should build on the present paper by incorporating related factors. This will enable researchers to evaluate the strength of weak and moderate interrelationships. Simultaneously, to ensure network stability and generalisability, researchers should test the network across various samples. Through this process, analyses will determine centrality indices. Although scholars have previously used NA in the field of individual differences and psychopathology, this approach is novel compared to established statistical methods. Consequently, reliability and fit tests are being developed. To ensure that findings are robust, investigators should conduct further analysis using complementary, established analytical techniques (e.g., latent variable analysis and multidimensional models; Prisciandaro and Roberts, 2009).

Consistent with Dagnall et al. (2022a), this study illustrates how the application of NA to PB research furthers psychopathological and clinical understanding. Moreover, NA can also enable researchers to test, refine, and advance emerging belief-based wellbeing models. From a design perspective, the use of a cross-sectional approach limits the generalisability of findings. Data collected at one point provides only a temporally restricted snapshot of the variables under observation. Consequently, despite highlighting nuanced associations between constructs, it is not possible to infer causation. This can only be achieved using experimental and/or longitudinal designs. Indeed, multiple measurements of factors allow investigators to establish stability in constructs and measurements, observe changes over time, and determine cause and effect. Correspondingly, the use of NA does not establish the direction of the relationship. Instead, the authors’ suppositions derive from inferences based on theoretical knowledge, previous research, and existing evidence, specifically that schizotypy is trait-based (see Ericson et al., 2011), PB is stable over time (Drinkwater et al., 2024), and investigators typically operationalize wellbeing as a consequence or outcome (Dagnall et al., 2022b).

Using Cohen’s (2013) guidelines for relationship strengths, several statistically significant correlations observed in this study were found to be negligible or small. While scholars frequently reference these guidelines, critics argue that they are overly stringent. One concern is that the suggested classifications are based on qualitative impressions rather than rigorous analysis. The authors acknowledged this and adopted the recommendations of Gignac and Szodorai (2016): 0.10 for small, 0.20 for medium, and 0.30 for large. These classifications were considered more appropriate as they were derived from large-scale meta-analyses of published results.

Since the study was exploratory in nature, readers should interpret the outcomes with caution. While some of the reported relationships align with those found in established literature (e.g., connections between PB, CT, and Schizotypy), others remain under-researched (i.e., variations in factors related to positive wellbeing based on belief type). Thus, researchers should conduct further studies to replicate and expand on the observed outcomes. This should involve considering believers as heterogeneous, especially whether specific Cognitive-Perceptual and affective personality factors affect the relationship between PB and wellbeing/psychological adjustment. Furthermore, future investigations should utilize a broader range of positive wellbeing measures. Although the researchers evaluated established wellbeing factors in the paper, the constructs examined represented only a limited array of psychological outcomes.

## Data Availability

Raw data supporting the conclusions of this article will be made available by the authors, without undue reservation.

## References

[ref1] Arndt S. Alliger R. J. Andreasen N. C. (1991). The distinction of positive and negative symptoms: the failure of a two-dimensional model. Br. J. Psychiatry 158, 317–322. doi: 10.1192/bjp.158.3.317 2036528

[ref2] Benjamini Y. Hochberg Y. (1995). Controlling the false discovery rate: a practical and powerful approach to multiple testing. J. R. Stat. Soc. B (Methodological) 57, 289–300. doi: 10.1111/j.2517-6161.1995.tb02031.x

[ref3] Bensley D. A. Lilienfeld S. O. Rowan K. A. Masciocchi C. M. Grain F. (2020). The generality of belief in unsubstantiated claims. Appl. Cogn. Psychol. 34, 16–28. doi: 10.1002/acp.3581

[ref4] Betsch T. Jäckel P. Hammes M. Brinkmann B. J. (2021). On the adaptive value of paranormal beliefs-a qualitative study. Integr. Psychol. Behav. Sci. 55, 318–328. doi: 10.1007/s12124-020-09594-5, PMID: 33464467 PMC7813974

[ref5] Borsboom D. Deserno M. K. Rhemtulla M. Epskamp S. Fried E. I. McNally R. J.. (2021). Network analysis of multivariate data in psychological science. Nat. Rev. Methods Primers 1, 1–18. doi: 10.1038/s43586-021-00055-w, PMID: 39934837

[ref6] Brotherton R. French C. C. Pickering A. D. (2013). Measuring belief in conspiracy theories: the generic Conspiracist beliefs scale. Front. Psychol. 4:279. doi: 10.3389/fpsyg.2013.00279, PMID: 23734136 PMC3659314

[ref7] Bruder M. Imhoff R. Kammerer N. (2013). The nature of conspiracy beliefs: distinguishing paranoia and false belief from conspiracy thinking. Soc. Psychol. Bull. 8, 1–15.

[ref8] Bryant R. A. Creamer M. O’Donnell M. Forbes D. McFarlane A. C. Silove D.. (2017). Acute and chronic posttraumatic stress symptoms in the emergence of posttraumatic stress disorder: a network analysis. JAMA Psychiatry 74, 135–142. doi: 10.1001/jamapsychiatry.2016.3470 28002832

[ref9] Carver C. S. (1997). You want to measure coping but your protocol’s too long: consider the brief cope. Int. J. Behav. Med. 4, 92–100. doi: 10.1207/s15327558ijbm0401_6, PMID: 16250744

[ref10] Chen J. Chen Z. (2008). Extended Bayesian information criteria for model selection with large model spaces. Biometrika 95, 759–771. doi: 10.1093/biomet/asn034

[ref11] Claridge G. (1997). Schizotypy: Implications for illness and health. Oxford: Oxford University Press.

[ref12] Cohen J. (2013). Statistical power analysis for the behavioral sciences. Abingdon: Routledge.

[ref13] Dagnall N. Denovan A. Drinkwater K. G. (2022a). Paranormal belief, cognitive-perceptual factors, and well-being: a network analysis. Front. Psychol. 13:967823. doi: 10.3389/fpsyg.2022.967823, PMID: 36186327 PMC9521162

[ref14] Dagnall N. Denovan A. Drinkwater K. G. (2022b). Variations in well-being as a function of paranormal belief and psychopathological symptoms: a latent profile analysis. Front. Psychol. 13:886369. doi: 10.3389/fpsyg.2022.886369, PMID: 35814073 PMC9263512

[ref15] Dagnall N. Denovan A. Drinkwater K. G. Escolà-Gascón Á. (2022c). Paranormal belief and well-being: the moderating roles of transliminality and psychopathology-related facets. Front. Psychol. 13:915860. doi: 10.3389/fpsyg.2022.915860, PMID: 36046418 PMC9421129

[ref16] Dagnall N. Denovan A. Drinkwater K. G. Escolà-Gascón A. (2023). The generic Conspiracist beliefs Scale-5: further psychometric evaluation using a United Kingdom-based sample. Front. Psychol. 14:838. doi: 10.3389/fpsyg.2023.1303838, PMID: 38094703 PMC10716305

[ref17] Dagnall N. Denovan A. Drinkwater K. Parker A. Clough P. (2017). Statistical bias and endorsement of conspiracy theories. Appl. Cogn. Psychol. 31, 368–378. doi: 10.1002/acp.3331

[ref18] Dagnall N. Drinkwater K. G. Denovan A. Gascón A. E. (2024). Variations in positive well-being as a function of the interaction between paranormal belief and schizotypy. Front. Psychol. 15:1396485. doi: 10.3389/fpsyg.2024.1396485, PMID: 39131861 PMC11310046

[ref19] Dagnall N. A. Drinkwater K. Parker A. Clough P. (2016). Paranormal experience, belief in the paranormal and anomalous beliefs. Paranthropology 7, 4–15.

[ref20] Dagnall N. Parker A. Munley G. (2007). Paranormal belief and reasoning. Personal. Individ. Differ. 43, 1406–1415. doi: 10.1016/j.paid.2007.04.017

[ref21] Dagnall N. Parker A. Munley G. Drinkwater K. (2010). Common paranormal belief dimensions. J. Sci. Explor. 24, 24, 477–494.

[ref22] Dakin B. C. Laham S. M. Tan N. P. J. Bastian B. (2021). Searching for meaning is associated with costly prosociality. PLoS One 16:e0258769. doi: 10.1371/journal.pone.0258769, PMID: 34695151 PMC8544877

[ref23] Denovan A. Dagnall N. Drinkwater K. Parker A. (2018). Latent profile analysis of schizotypy and paranormal belief: associations with probabilistic reasoning performance. Front. Psychol. 9:35. doi: 10.3389/fpsyg.2018.00035, PMID: 29434562 PMC5791384

[ref24] Diener E. D. Emmons R. A. Larsen R. J. Griffin S. (1985). The satisfaction with life scale. J. Pers. Assess. 49, 71–75. doi: 10.1207/s15327752jpa4901_13 16367493

[ref25] Drinkwater K. G. Dagnall N. Denovan A. Williams C. (2021a). Paranormal belief, thinking style and delusion formation: a latent profile analysis of within-individual variations in experience-based paranormal facets. Front. Psychol. 12:670959. doi: 10.3389/fpsyg.2021.670959, PMID: 34262510 PMC8273333

[ref26] Drinkwater K. G. Dagnall N. Denovan A. Williams C. (2021b). Differences in cognitive-perceptual factors arising from variations in self-professed paranormal ability. Front. Psychol. 12:681520. doi: 10.3389/fpsyg.2021.681520 34177736 PMC8222626

[ref27] Drinkwater K. Dagnall N. Parker A. (2012). Reality testing, conspiracy theories, and paranormal beliefs. J. Parapsychol. 76, 57–77.

[ref28] Drinkwater K. G. Denovan A. Dagnall N. (2024). Paranormal belief, psychopathological symptoms, and well-being: latent profile analysis and longitudinal assessment of relationships. PLoS One 19:e0297403. doi: 10.1371/journal.pone.0297403, PMID: 38446771 PMC10917279

[ref29] Drinkwater K. Denovan A. Dagnall N. Parker A. (2017). An assessment of the dimensionality and factorial structure of the revised paranormal belief scale. Front. Psychol. 8:1693. doi: 10.3389/fpsyg.2017.01693, PMID: 29018398 PMC5622942

[ref30] Dyrendal A. Kennair L. E. O. Bendixen M. (2021). Predictors of belief in conspiracy theory: the role of individual differences in schizotypal traits, paranormal beliefs, social dominance orientation, right wing authoritarianism and conspiracy mentality. Personal. Individ. Differ. 173:110645. doi: 10.1016/j.paid.2021.110645

[ref31] Eisenberg S. A. Shen B. J. Schwarz E. R. Mallon S. (2012). Avoidant coping moderates the association between anxiety and patient-rated physical functioning in heart failure patients. J. Behav. Med. 35, 253–261. doi: 10.1007/s10865-011-9358-0, PMID: 21660588

[ref32] Epskamp S. Borsboom D. Fried E. I. (2018). Estimating psychological networks and their accuracy: a tutorial paper. Behav. Res. Methods 50, 195–212. doi: 10.3758/s13428-017-0862-1, PMID: 28342071 PMC5809547

[ref33] Ericson M. Tuvblad C. Raine A. Young-Wolff K. Baker L. A. (2011). Heritability and longitudinal stability of schizotypal traits during adolescence. Behav. Genet. 41, 499–511. doi: 10.1007/s10519-010-9401-x, PMID: 21369821 PMC3123391

[ref34] Fitzpatrick O. D. Shook S. L. (1994). Belief in the paranormal: does identity development during the college years make a difference? An initial investigation. J. Parapsychol. 58, 315–330.

[ref35] Fladerer M. P. Braun S. (2020). Managers’ resources for authentic leadership–a multi-study exploration of positive psychological capacities and ethical organizational climates. Br. J. Manag. 31, 325–343. doi: 10.1111/1467-8551.12396

[ref36] Friedman J. Hastie T. Tibshirani R. (2008). Sparse inverse invariance estimation with the graphical lasso. Biostatistics 9, 432–441. doi: 10.1093/biostatistics/kxm045, PMID: 18079126 PMC3019769

[ref37] Gignac G. E. Szodorai E. T. (2016). Effect size guidelines for individual differences researchers. Personal. Individ. Differ. 102, 74–78. doi: 10.1016/j.paid.2016.06.069

[ref38] Goertzel T. (1994). Belief in conspiracy theories. Polit. Psychol. 15:731. doi: 10.2307/3791630, PMID: 39964225

[ref39] Grigoryev D. Gallyamova A. (2023). Social worldviews predict the general factor of paranormal and generic conspiracist beliefs. Span. J. Psychol. 26:e19. doi: 10.1017/SJP.2023.18, PMID: 37357156

[ref40] Hair J. F. Hult G. T. M. Ringle C. M. Sarstedt M. (2022). A primer on partial least squares structural equation modeling (PLS-SEM). 3rd Edn. Thousand Oaks, CA: Sage.

[ref41] Heshmati S. Oravecz Z. Brick T. R. Roeser R. W. (2020). Assessing psychological well-being in early adulthood: empirical evidence for the structure of daily well-being via network analysis. Appl. Dev. Sci. 26, 207–225. doi: 10.31234/osf.io/6cyfw

[ref42] Imhoff R. Bruder M. (2014). Speaking (un-) truth to power: conspiracy mentality as a generalized political attitude. Eur. J. Personal. 28, 25–43. doi: 10.1002/per.1930

[ref43] Irwin H. J. (2009). The psychology of paranormal belief: A researcher’s handbook. Hatfield: University of Hertfordshire Press.

[ref44] Irwin H. J. Dagnall N. Drinkwater K. (2012a). Paranormal belief and biases in reasoning underlying the formation of delusions. Aust. J. Parapsychol. 12, 7–21.

[ref45] Irwin H. J. Dagnall N. Drinkwater K. (2012b). Paranormal beliefs and cognitive processes underlying the formation of delusions. Aust. J. Parapsychol. 12, 107–126.

[ref46] Jones P. J. Mair P. McNally R. J. (2018). Visualizing psychological networks: a tutorial in R. Front. Psychol. 9:1742. doi: 10.3389/fpsyg.2018.01742, PMID: 30283387 PMC6156459

[ref47] Kay C. S. Slovic P. (2023). The generic Conspiracist beliefs scale–5: a short-form measure of conspiracist ideation. J. Res. Pers. 102:104315. doi: 10.1016/j.jrp.2022.104315

[ref48] Kees J. Berry C. Burton S. Sheehan K. (2017). An analysis of data quality: professional panels, student subject pools, and Amazon's mechanical Turk. J. Advert. 46, 141–155. doi: 10.1080/00913367.2016.1269304

[ref49] Kennedy J. E. Kanthamani H. (1995). An exploratory study of the effects of paranormal and spiritual experiences on peoples’ lives and well-being. J. Am. Soc. Psych. Res. 89, 249–264.

[ref50] Krishnaveni R. Deepa R. (2013). Controlling common method variance while measuring the impact of emotional intelligence on well-being. Vikalpa 38, 41–48. doi: 10.1177/0256090920130104

[ref51] Kruglanski A. W. Webster D. M. (1996). Motivated closing of the mind: "seizing" and "freezing.". Psychol. Rev. 103, 263–283. doi: 10.1037/0033-295X.103.2.263, PMID: 8637961

[ref52] Lawrence E. Peters E. (2004). Reasoning in believers in the paranormal. J. Nerv. Ment. Dis. 192, 727–733. doi: 10.1097/01.nmd.0000144691.22135.d0, PMID: 15505516

[ref53] Leman P. J. Cinnirella M. (2007). A major event, a minor event, and a conspiracy theory: the influence of emotional and cognitive factors on the endorsement of conspiracy theories. Personal. Individ. Differ. 9, 18–28. doi: 10.53841/bpsspr.2007.9.2.18

[ref54] Levinson C. A. Zerwas S. Calebs B. Forbush K. Kordy H. Watson H.. (2017). The core symptoms of bulimia nervosa, anxiety, and depression: a network analysis. J. Abnorm. Psychol. 126, 340–354. doi: 10.1037/abn0000254, PMID: 28277735 PMC5378619

[ref55] Lindeman M. Svedholm-Häkkinen A. M. Lipsanen J. (2015). Ontological confusions but not mentalizing abilities predict religious belief, paranormal belief, and belief in supernatural purpose. Cognition 134, 63–76. doi: 10.1016/j.cognition.2014.09.008, PMID: 25460380

[ref56] Lobato E. Mendoza J. Sims V. Chin M. (2014). Examining the relationship between conspiracy theories, paranormal beliefs, and pseudoscience acceptance among a university population. Appl. Cogn. Psychol. 28, 617–625. doi: 10.1002/acp.3042

[ref57] Maraldi E. D. O. (2014). Medium or author? A preliminary model relating dissociation, paranormal belief systems and self-esteem. J. Soc. Psych. Res. 78, 1–24.

[ref58] Newman D. B. Nezlek J. B. Thrash T. M. (2018). The dynamics of searching for meaning and presence of meaning in daily life. J. Pers. 86, 368–379. doi: 10.1111/jopy.12321, PMID: 28423186

[ref59] Newman D. B. Schneider S. Stone A. A. (2022). Contrasting effects of finding meaning and searching for meaning, and political orientation and religiosity, on feelings and behaviors during the COVID-19 pandemic. Personal. Soc. Psychol. Bull. 48, 923–936. doi: 10.1177/01461672211030383, PMID: 34238068

[ref60] Oliver J. E. Wood T. J. (2018). Enchanted America: How intuition and reason divide our politics. Chicago, IL: University of Chicago Press.

[ref61] Parra A. Corbetta J. M. (2014). Changes resulting from paranormal/spiritual experiences and their effects on people’s wellbeing: an exploratory examination. J. Study Spiritual. 4, 73–82. doi: 10.1179/2044024314Z.00000000022

[ref62] Pérez Navarro J. M. Martínez Guerra X. (2020). Personality, cognition, and morbidity in the understanding of paranormal belief. PsyCh J. 9, 118–131. doi: 10.1002/pchj.295 31183994

[ref63] Prisciandaro J. J. Roberts J. E. (2009). A comparison of the predictive abilities of dimensional and categorical models of unipolar depression in the national comorbidity survey. Psychol. Med. 39, 1087–1096. doi: 10.1017/S0033291708004522, PMID: 18845012

[ref64] Raine A. Benishay D. (1995). The SPQ-B: a brief screening instrument for schizotypal personality disorder. J. Personal. Disord. 9, 346–355. doi: 10.1521/pedi.1995.9.4.346

[ref65] Rizeq J. Flora D. B. Toplak M. E. (2021). An examination of the underlying dimensional structure of three domains of contaminated mindware: paranormal beliefs, conspiracy beliefs, and anti-science attitudes. Think. Reason. 27, 187–211. doi: 10.1080/13546783.2020.1759688

[ref66] Rosenberg M. (1965). Society and the adolescent self-image. Princeton, NJ: Princeton University Press.

[ref67] Russo-Netzer P. Icekson T. (2023). An underexplored pathway to life satisfaction: the development and validation of the synchronicity awareness and meaning-detecting scale. Front. Psychol. 13:1053296. doi: 10.3389/fpsyg.2022.1053296, PMID: 36726512 PMC9885050

[ref68] Seligman M. E. P. (2011). Flourish: A visionary new understanding of happiness and well-being. New York: Penguin Random House Australia.

[ref69] Spector P. E. (2019). Do not cross me: optimizing the use of cross-sectional designs. J. Bus. Psychol. 34, 125–137. doi: 10.1007/s10869-018-09613-8

[ref70] Stanovich K. E. Toplak M. E. West R. F. (2008). “The development of rational thought: a taxonomy of heuristics and biases” in Advances in child development and behavior. ed. Kail R. V., vol. 36 (Amsterdam, Netherlands: Elsevier Academic Press), 251–285. 10.1016/s0065-2407(08)00006-2 18808045

[ref71] Steger M. F. (2009). “Meaning in life” in Oxford handbook of positive psychology. ed. Lopez S. J.. 2nd ed (Oxford, UK: Oxford University Press), 679–687.

[ref72] Steger M. (2017). “Meaning in life and wellbeing” in Wellbeing, recovery and mental health. eds. Slade M. Oades L. Jarden A. (Cambridge: Cambridge University Press), 75–85.

[ref73] Steger M. F. Frazier P. Oishi S. Kaler M. (2006). The meaning in life questionnaire: assessing the presence of and search for meaning in life. J. Couns. Psychol. 53, 80–93. doi: 10.1037/0022-0167.53.1.80

[ref74] Tobacyk J. J. (2004). A revised paranormal belief scale. Int. J. Transpers. Stud. 23, 94–98. doi: 10.24972/ijts.2004.23.1.94

[ref75] Unterrassner L. Wyss T. A. Wotruba D. Ajdacic-Gross V. Haker H. Rössler W. (2017). Psychotic-like experiences at the healthy end of the psychosis continuum. Front. Psychol. 8:775. doi: 10.3389/fpsyg.2017.00775, PMID: 28555120 PMC5431212

[ref76] Valente T. W. (2012). Network interventions. Science 337, 49–53. doi: 10.1126/science.1217330 22767921

[ref77] Van Prooijen J. W. Acker M. (2015). The influence of control on belief in conspiracy theories: conceptual and applied extensions. Appl. Cogn. Psychol. 29, 753–761. doi: 10.1002/acp.3161

[ref78] Van Prooijen J. W. Douglas K. M. (2017). Conspiracy theories as part of history: the role of societal crisis situations. Mem. Stud. 10, 323–333. doi: 10.1177/1750698017701615, PMID: 29081831 PMC5646574

[ref79] Williams C. Denovan A. Drinkwater K. Dagnall N. (2022). Thinking style and paranormal belief: the role of cognitive biases. Imagin. Cogn. Pers. 41, 274–298. doi: 10.1177/02762366211036435

[ref80] Williams E. Roberts B. L. H. (2016). The relationship between paranormal belief and the HEXACO domains of personality. J. Empir. Theol. 29, 212–238. doi: 10.1163/15709256-12341341

