# Supplementary material for: Paranormal belief, conspiracy endorsement, and positive wellbeing: a network analysis
Source: Front Psychol. 2025 Mar 10;16:1448067. doi: 10.3389/fpsyg.2025.1448067 (PMC11931579; doi:10.3389/fpsyg.2025.1448067)
Supplement: Supplementary file 1 [file Data_Sheet_1.docx]

Appendix S1. Descriptive statistics for study variables

| Variable | Mean | *SD* | Skewness | Kurtosis |
| --- | --- | --- | --- | --- |
| Paranormal Belief | 88.95 | 34.81 | -.03 | -.84 |
| Conspiracy Endorsement | 15.19 | 4.89 | -.18 | -.57 |
| Cognitive-Perceptual | 2.58 | 2.30 | .63 | -.60 |
| Interpersonal | 4.02 | 2.57 | -.03 | -.71 |
| Disorganized | 1.64 | 1.85 | .92 | -.33 |
| Meaning in Life Presence | 21.63 | 6.89 | -.36 | -.17 |
| Meaning in Life Search | 20.97 | 7.10 | -.32 | -.28 |
| Active Coping | 31.80 | 7.77 | -.22 | -.31 |
| Avoidant Coping | 22.31 | 7.71 | .86 | .10 |
| Self-esteem | 27.89 | 6.32 | -.21 | -.18 |
| Satisfaction with Life | 20.34 | 7.79 | -.22 | -.69 |
