# Supplementary material for: Paranormal belief, conspiracy endorsement, and positive wellbeing: a network analysis
Source: Front Psychol. 2025 Mar 10;16:1448067. doi: 10.3389/fpsyg.2025.1448067 (PMC11931579; doi:10.3389/fpsyg.2025.1448067)
Supplement: Supplementary file 2 [file Data_Sheet_2.docx]

Appendix S2. Weights matrix from the network analysis

| Variable | 1 | 2 | 3 | 4 | 5 | 6 | 7 | 8 | 9 | 10 | 11 |
| --- | --- | --- | --- | --- | --- | --- | --- | --- | --- | --- | --- |
| 1. Paranormal Belief |  |  |  |  |  |  |  |  |  |  |  |
| 1. Conspiracy Endorsement | .40 |  |  |  |  |  |  |  |  |  |  |
| 1. Cognitive-Perceptual | .29 | .16 |  |  |  |  |  |  |  |  |  |
| 1. Interpersonal | -.05 | .00 | .14 |  |  |  |  |  |  |  |  |
| 1. Disorganized | -.02 | .00 | .34 | .45 |  |  |  |  |  |  |  |
| 1. Meaning in Life Presence | .11 | .00 | .03 | -.06 | .00 |  |  |  |  |  |  |
| 1. Meaning in Life Search | .17 | .07 | .07 | .00 | .00 | .03 |  |  |  |  |  |
| 1. Active Coping | -.03 | -.01 | .09 | .00 | .00 | .19 | .16 |  |  |  |  |
| 1. Avoidant Coping | .15 | .03 | .06 | .00 | .10 | .00 | .17 | .07 |  |  |  |
| 1. Self-esteem | -.01 | .00 | .00 | -.09 | -.11 | .35 | -.12 | .19 | -.26 |  |  |
| 1. Satisfaction with Life | .00 | .00 | -.02 | -.07 | .02 | .38 | -.08 | .00 | .10 | .25 |  |
