# Supplementary material for: Paranormal belief, conspiracy endorsement, and positive wellbeing: a network analysis
Source: Front Psychol. 2025 Mar 10;16:1448067. doi: 10.3389/fpsyg.2025.1448067 (PMC11931579; doi:10.3389/fpsyg.2025.1448067)
Supplement: Supplementary file 3 [file Data_Sheet_3.pdf]

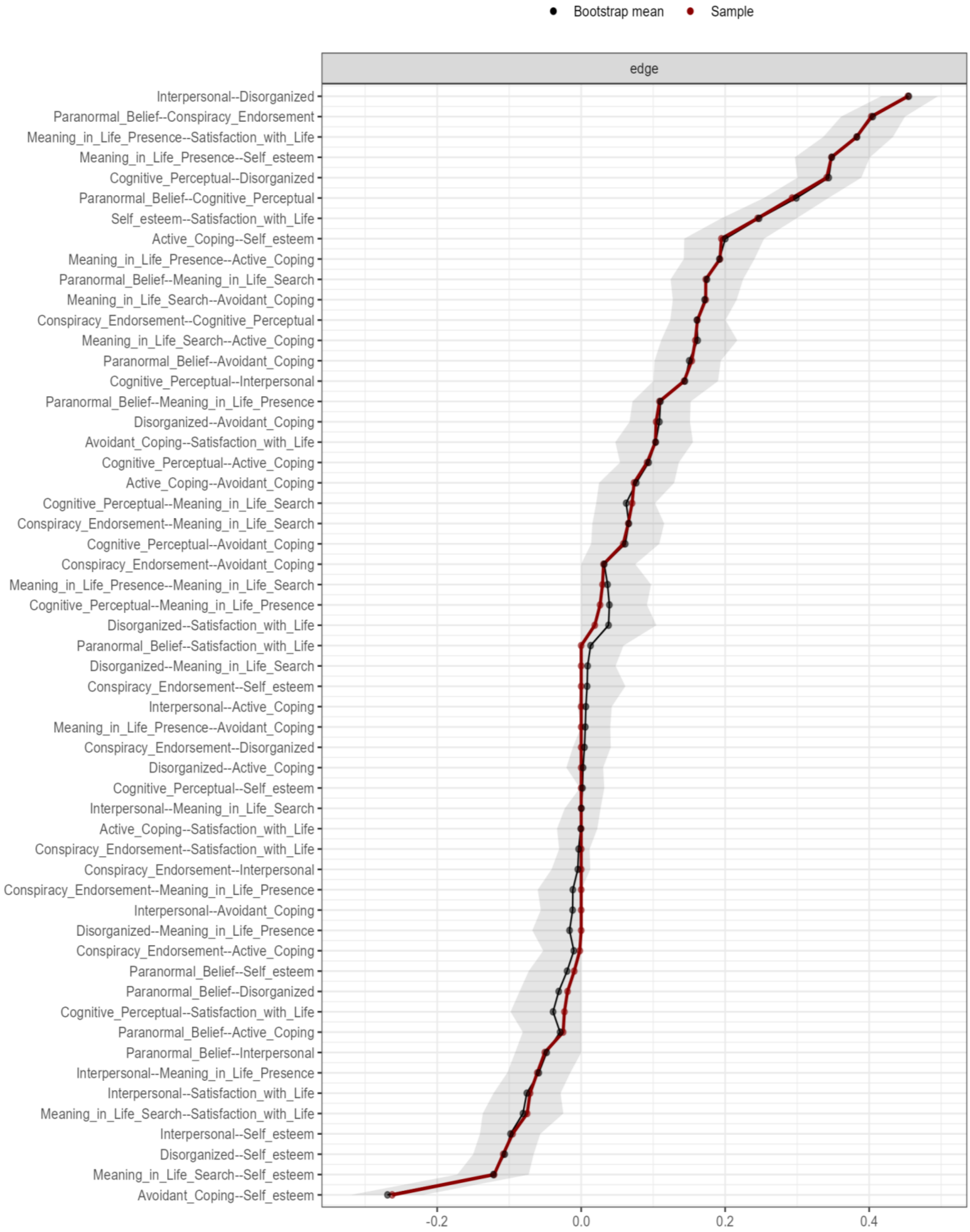

Appendix S3. Bootstrapped confidence intervals (CIs) of the network analysis edge weights.
Note. Edge weight values depicted by the red line; 95% CIs represented by the grey area.
