# Supplementary material for: Paranormal belief, conspiracy endorsement, and positive wellbeing: a network analysis
Source: Front Psychol. 2025 Mar 10;16:1448067. doi: 10.3389/fpsyg.2025.1448067 (PMC11931579; doi:10.3389/fpsyg.2025.1448067)

Results

Network Analysis

Summary of Network

Number of nodes	Number of non-zero edges	Sparsity
11	36 / 55	0.345

Centrality measures per variable

	Network			
Variable	Betweenness	Closeness	Strength	Expected influence
Traditional_Religious_Belief	0.925	1.604	-0.169	0.293
Conspiracy_Endorsement	-0.856	-0.644	-1.351	-0.021
Cognitive_Perceptual	0.925	0.315	1.102	1.845
Interpersonal	-0.856	-1.254	-0.716	-1.038
Disorganized	0.671	-0.322	0.434	0.381
Meaning_in_Life_Presence	1.179	1.165	1.002	1.422
Meaning_in_Life_Search	-0.856	-0.687	-0.324	-0.371
Active_Coping	-0.856	-1.042	-1.261	0.049
Avoidant_Coping	-0.856	0.224	-0.010	-0.662
Self_esteem	1.434	1.337	1.807	-1.615
Satisfaction_with_Life	-0.856	-0.696	-0.514	-0.282

Weights matrix

	Network										
Variable	Traditional_Religious_Belief	Conspiracy_Endorsement	Cognitive_Perceptual	Interpersonal	Disorganized	Meaning_in_Life_Presence	Meaning_in_Life_Search	Active_Coping	Avoidant_Coping	Self_esteem	Satisfaction_with_Life
Traditional_Religious_Belief	0.000	0.198	0.169	0.000	-0.046	0.192	0.166	0.000	0.102	-0.043	0.000
Conspiracy_Endorsement	0.198	0.000	0.265	0.000	0.000	0.000	0.108	0.000	0.074	0.000	0.000
Cognitive_Perceptual	0.169	0.265	0.000	0.120	0.356	0.020	0.100	0.081	0.090	0.000	-0.006
Interpersonal	0.000	0.000	0.120	0.000	0.448	-0.057	0.000	0.000	0.000	-0.100	-0.066
Disorganized	-0.046	0.000	0.356	0.448	0.000	0.000	0.000	0.000	0.105	-0.099	0.000
Meaning_in_Life_Presence	0.192	0.000	0.020	-0.057	0.000	0.000	0.004	0.191	0.000	0.345	0.374
Meaning_in_Life_Search	0.166	0.108	0.100	0.000	0.000	0.004	0.000	0.147	0.185	-0.107	-0.062
Active_Coping	0.000	0.000	0.081	0.000	0.000	0.191	0.147	0.000	0.062	0.185	0.000
Avoidant_Coping	0.102	0.074	0.090	0.000	0.105	0.000	0.185	0.062	0.000	-0.248	0.086
Self_esteem	-0.043	0.000	0.000	-0.100	-0.099	0.345	-0.107	0.185	-0.248	0.000	0.242
Satisfaction_with_Life	0.000	0.000	-0.006	-0.066	0.000	0.374	-0.062	0.000	0.086	0.242	0.000

Network

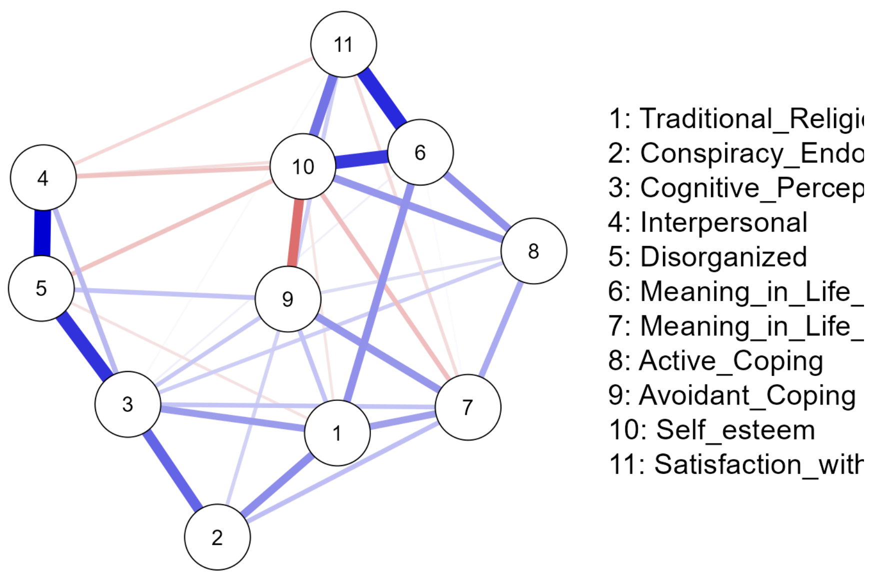

Centrality Plot

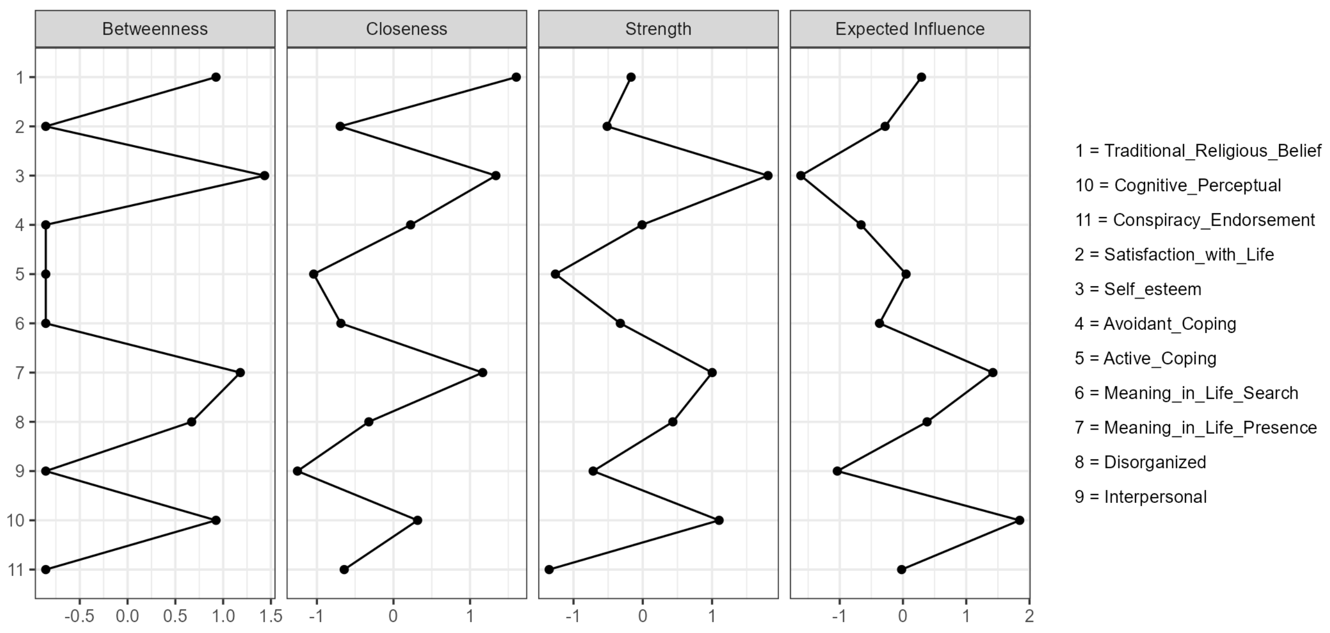

Bootstrap summary of Network

Type	Number of bootstraps
Nonparametric	1000

Edge Stability

Network

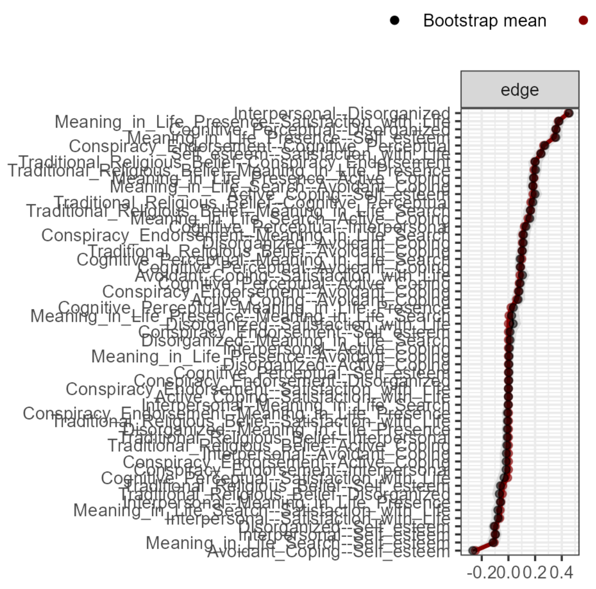

Centrality Stability

Network

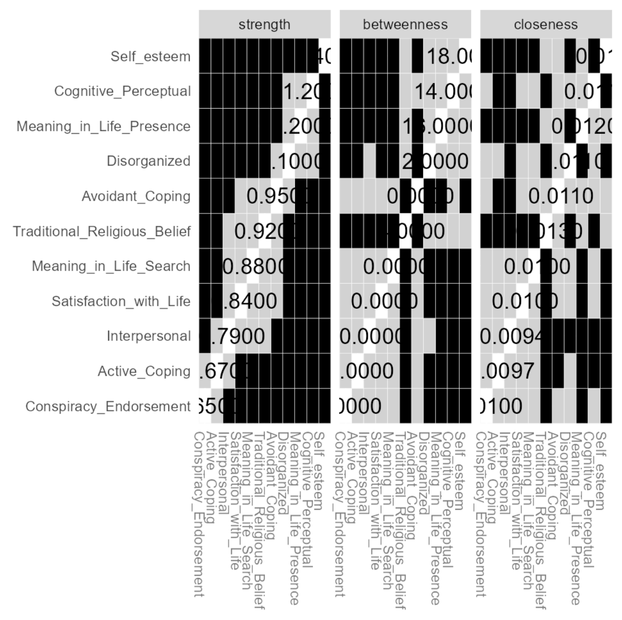

Supplement: Supplementary file 4 [file Data_Sheet_4.pdf]
